# A Scalable Haze‐Free Antireflective Hierarchical Surface with Self‐Cleaning Capability

**DOI:** 10.1002/advs.202202781

**Published:** 2022-07-28

**Authors:** Seungtae Oh, Jin‐Woo Cho, Jihun Lee, Jeonghoon Han, Sun‐Kyung Kim, Youngsuk Nam

**Affiliations:** ^1^ Carbon Neutral Technology R&D Department Korea Institute of Industrial Technology (KITECH) Cheonan 31056 Republic of Korea; ^2^ Department of Applied Physics Kyung Hee University Yongin 17104 Republic of Korea; ^3^ Department of Mechanical Engineering Pohang University of Science and Technology (POSTECH) Pohang 37673 Republic of Korea; ^4^ Department of Mechanical Engineering Korea Advanced Institute of Science and Technology (KAIST) Daejeon 34141 Republic of Korea

**Keywords:** antireflective, hierarchical structure, scattering suppression, self‐cleaning, superhydrophobic

## Abstract

The lotus effect indicates that a superhydrophobic, self‐cleaning surface can be obtained by roughening the topography of a hydrophobic surface. However, attaining high transmittance and clarity through a roughened surface remains challenging because of its strong scattering characteristics. Here, a haze‐free, antireflective superhydrophobic surface that consists of hierarchically designed nanoparticles is demonstrated. Close‐packed, deep‐subwavelength‐scale colloidal silica nanoparticles and their upper, chain‐like fumed silica nanoparticles individually fulfill haze‐free broadband antireflection and self‐cleaning functions. These double‐layered hierarchical surfaces are obtained via a scalable spraying process that permits precise control over the coating morphology to attain the desired optical and wetting properties. They provide a “specular” visible transmittance of >97% when double‐side coated and a record‐high self‐cleaning capability with a near‐zero sliding angle. Self‐cleaning experiments on photovoltaic devices verify that the developed surfaces can significantly enhance power conversion efficiencies and aid in retaining pristine device performance in a dusty environment.

## Graphical abstract

Bumpy morphology of superhydrophobic coatings scatters incident light, thereby lowering transparency. Here, broadband, haze‐free, and antireflective surfaces with self‐cleaning capability is reported. “Hierarchically” designed coatings provide large “specular” transmittance (>97%) in visible and self‐cleaning capability. As a proof‐of‐concept, experiments are conducted on photovoltaic devices in dusty environment and observed a complete recovery of performance with improved (>6%) power conversion efficiency.

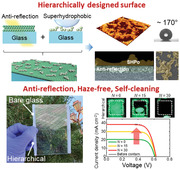

## 1 Introduction

Superhydrophobic surfaces offer an exceptional opportunity for regulating diverse interfacial phenomena through their extreme liquid repellence. On superhydrophobic surfaces, water droplets retain a spherical shape (static contact angle, *θ*_s_ > 150°) and can easily roll down when the surfaces are tilted by only a few degrees (sliding angle, *θ*_sa_ < 10°).^[^ ^1^, ^2^ ^]^ One facile strategy for obtaining superhydrophobicity (*θ*_s_ > 150° and *θ*_sa_ < 10°)^[^ ^1^, ^3^, ^4^, ^5^ ^]^ is to roughen hydrophobic surfaces,^[^ ^1^ ^]^ which is often referred to as the lotus effect.^[^ ^2^ ^]^ The *θ*_s_ of water droplets on flat hydrophobic surfaces is typically 100°–120° solely because of their low surface chemical energy, but superhydrophobicity can be achieved only when the surfaces are structured at the micro‐^[^ ^6^, ^7^ ^]^ or nanoscales.^[^ ^8^, ^9^, ^10^, ^11^, ^12^, ^13^, ^14^, ^15^, ^16^, ^17^, ^18^, ^19^, ^20^, ^21^, ^22^, ^23^, ^24^, ^25^, ^26^, ^27^, ^28^, ^29^ ^]^ Superhydrophobic surfaces can trap air beneath water droplets (i.e., Cassie state),^[^ ^30^ ^]^ which offers a means to promptly eliminate contaminants and to avoid icing and fouling in various environmentally benign.^[^ ^6^, ^7^, ^8^, ^9^, ^10^, ^11^, ^12^, ^13^, ^14^, ^15^, ^16^, ^17^, ^18^, ^19^, ^20^, ^21^, ^22^, ^23^, ^24^, ^25^, ^26^, ^27^, ^28^, ^29^ ^]^ To date, high‐transparency (i.e., high “specular” transmittance) superhydrophobic coatings are essential for developing low‐maintenance glass‐based applications, such as smart windows,^[^ ^31^ ^]^ touch panels,^[^ ^32^ ^]^ see‐through displays,^[^ ^33^ ^]^ LiDARs^[^ ^8^ ^]^ and photovoltaics (PVs),^[^ ^34^, ^35^, ^36^ ^]^ to name a few. Note that we define the term transparency as an optical quantity of being transparent and “haze‐free” such that it is distinguished from the amount of light simply passing from one medium to another (i.e., transmittance).^[^ ^37^ ^]^ Hence, high‐transparency surfaces allow light to pass through them with marginal scattering and reflection losses, thereby providing a clear vision.^[^ ^38^ ^]^ Therefore, they are characterized by low haze and high transmittance, which is a prerequisite for glass‐based applications.

**Figure 1:**
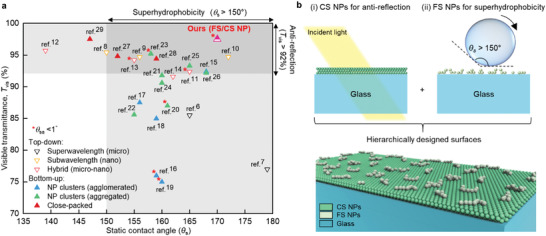
High‐transparency superhydrophobic surfaces. a) Previously reported *T*_vis_ and *θ*_s_ values.^[^ ^6^, ^7^, ^8^, ^9^, ^10^, ^11^, ^12^, ^13^, ^14^, ^15^, ^16^, ^17^, ^18^, ^19^, ^20^, ^21^, ^22^, ^23^, ^24^, ^25^, ^26^, ^27^, ^28^, ^29^ ^]^ The asterisk denotes the results with *θ*_sa_ < 1˚. *T*_vis_ was obtained by averaging the transmittance acquired at each wavelength (380–780 nm) that was extracted from the transmittance spectra reported,^[^ ^6^, ^7^, ^8^, ^9^, ^10^, ^11^, ^12^, ^13^, ^14^, ^15^, ^16^, ^17^, ^18^, ^19^, ^20^, ^21^, ^22^, ^23^, ^24^, ^25^, ^26^, ^27^, ^28^, ^29^ ^]^ weighted by the AM 1.5G solar. b) Schematics illustrating a hierarchically designed surface that consists of close‐packed colloidal silica (CS) NPs (i) and fumed silica (FS) NPs (ii).

Recent studies on superhydrophobic surfaces have been based on top‐down texturing^[^ ^6^, ^7^, ^8^, ^9^, ^10^, ^11^, ^12^, ^13^, ^14^ ^]^ and bottom‐up nanoparticle (NP) coating processes.^[^ ^15^, ^16^, ^17^, ^18^, ^19^, ^20^, ^21^, ^22^, ^23^, ^24^, ^25^, ^26^, ^27^, ^28^, ^29^ ^]^ Quantitative comparison of our work with previous superhydrophobic coatings is performed in terms of visible (380–780 nm) transmittance (*T*_vis_) and static contact angle (*θ*_s_) as shown in **Figure** **1**a; and Table S1, Supporting Information. The top‐down approach,^[^ ^6^, ^7^, ^8^, ^9^, ^10^, ^11^, ^12^, ^13^, ^14^ ^]^ in which visibly transparent surfaces are textured above (i.e., superwavelength)^[^ ^6^, ^7^ ^]^ or below (i.e., subwavelength)^[^ ^8^, ^9^, ^10^ ^]^ wavelength scale by means of various etching techniques with^[^ ^7^, ^8^, ^9^ ^]^ or without^[^ ^6^, ^10^ ^]^ a prior lithography step, have successfully provided *θ*_s_ of >150°. For superwavelength schemes,^[^ ^6^, ^7^ ^]^ the bumpy topology scatters incident light in both the forward and backward directions, thereby degrading the visible transmittance (<86% when applied on glass substrates, as shown in Figure 1a) and vision resolution. In comparison, subwavelength superhydrophobic schemes have been attempted to focus mainly on improving the transmittance.^[^ ^8^, ^9^, ^10^ ^]^ For example, Infante et al. constructed randomly distributed nanopillars (80–120 nm in diameter) using reactive ion etching with copper nanoparticles on glass substrates.^[^ ^9^ ^]^ Although this approach provided an enhanced visible transmittance of ≈94%, each nanopillar, which was loosely packed in the array, acted as a Mie scattering‐mediated optical antenna, thus imposing a fundamental limit on the level of visual clarity. Moreover, such top‐down texturing approaches are not scalable, lack compatibility with various substrates, or involve complicated and costly fabrication techniques; thus, their potential application is limited.

The bottom‐up approach treats low‐refractive‐index (e.g., SiO_2_ and MgF_2_) NPs with high‐temperature calcination,^[^ ^15^, ^18^ ^]^ dipping,^[^ ^19^, ^20^, ^21^, ^22^, ^23^, ^26^ ^]^ spin‐coating,^[^ ^27^, ^28^ ^]^ and spraying processes^[^ ^16^, ^17^, ^19^, ^24^, ^25^ ^]^ and is more promising owing to its scalability, compatibility, and cost‐effectiveness. Furthermore, low‐haze surfaces, which promise the implementation of high‐resolution displays, can be readily obtained using deep‐subwavelength‐scale (<100 nm) NPs.^[^ ^39^ ^]^ However, achieving high‐transparency superhydrophobic surfaces based on NPs is nontrivial because the majority of previous studies used agglomerated^[^ ^15^, ^16^, ^17^, ^18^, ^19^ ^]^ or aggregated NP clusters^[^ ^20^, ^21^, ^22^, ^23^, ^24^, ^25^, ^26^ ^]^ to render superhydrophobicity. For example, Wong et al. constructed inverted micrometer (0.6–7 µm in height) cones that were composed of agglomerated 30 nm diameter SiO_2_ NPs, thus exhibiting root‐mean‐square surface roughness (*r*_q_) values of 1–10 µm.^[^ ^19^ ^]^ Therefore, they satisfied the criteria for superhydrophobicity (*θ*_s_≈150° and *θ*_sa_ < 10°) owing to the microscale *r*_q_ values accompanied by NP clusters; however, their visible transmittance values were significantly reduced relative to the uncoated structures. In principle, superhydrophobic surfaces with high “specular” transmittance can be achieved only when densely packed deep‐subwavelength‐scale NPs or pores are used.^[^ ^27^, ^28^, ^29^ ^]^ To this end, Yildirim et al. used three layers of close‐packed silica NPs with distinct porosity values,^[^ ^28^ ^]^ and Manabe et al. used ten alternating layers of close‐packed polymer nanofibers and silica NPs.^[^ ^27^ ^]^ As a result, both structures exhibited ≈95% visible transmittance; however, their *θ*_sa_ values exceeded 10° and 40°, respectively, because of the large droplet contact line depinning force associated with the small average pitch between the nanoparticles,^[^ ^4^ ^]^ which negates a self‐cleaning function on nearly planar devices. Note that a sliding angle of >10° is beyond the commonly accepted threshold for self‐cleaning.^[^ ^40^ ^]^

To summarize, all the previous strategies for superhydrophobic coatings are difficult to provide a haze‐free, broadband antireflective surface via a scalable and facile fabrication, which limits their use in practical high‐transparency devices. In particular, to impart an antireflection function to superhydrophobic coatings, their thickness and refractive index must be finely matched with the wavelength of incident light and must be uniform over the entire coated area. Furthermore, when NPs are used for superhydrophobic coatings, the scale of NPs should be smaller than approximately one‐tenth of the reference wavelength to retain haze‐free characteristic.^[^ ^39^ ^]^ To address these issues, in this study, we developed a scalable, high‐precision‐morphology superhydrophobic coating that reduces the surface reflectance without degrading visual clarity (Figure 1b). The rationale for this technology is based on “hierarchically” designed surfaces in which each constituent part acts independently to provide haze‐free broadband antireflection and self‐cleaning functions (Figure 1b). This design strategy is a key breakthrough because there is a fundamental trade‐off between the optical and wetting properties; a roughened surface is desirable for rendering superhydrophobicity, which adversely affects vision resolution. The developed superhydrophobic surfaces exhibit exceptional droplet mobility such that their self‐cleaning capability is fast and effective for various sizes of contaminants as compared to uncoated glass and commercial coatings. The optical properties of high transmittance and high clarity are valid over a broad range of wavelengths and incident angles, which enables high‐performance solar energy and display applications. As a proof‐of‐concept, we conducted experiments on PV devices in a dusty environment and observed improved power conversion efficiency (PCE) and a complete recovery of device performance using the hierarchically designed surfaces developed herein.

## 2 Results and Discussion

### 2.1 Fabrication of Hierarchically Designed Antireflective Superhydrophobic Surfaces

**Figure 2:**
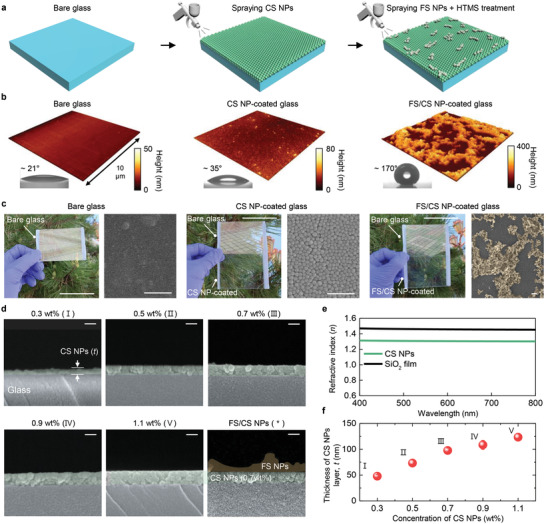
Fabrication of hierarchically designed, antireflective, and superhydrophobic surfaces. a) Schematics describing the fabrication steps for FS/CS NP coated surfaces. b) Atomic force microscope (AFM) images obtained at each fabrication step. Insets: photo images showing the water contact angle at each fabrication step. c) Photo and SEM images obtained at each fabrication step. The false‐colored areas indicate the FS NP coating. Scale bars: 5 cm and 200 nm for photo and SEM images, respectively. d) Cross‐sectional SEM images of CS NP coatings for increasing concentration, labeled I to V. The asterisk denotes a FS/CS NP coating. The green‐ and yellow false‐colored areas indicate CS NPs and FS NPs, respectively; Scale bars: 100 nm. e) Measured refractive index of *n* of the CS NPs and SiO_2_ film, respectively. f) Measured coating thickness of CS NPs for different concentrations. Each error bar represents the standard deviation from three independent measurements at three distinct positions on three separate samples.

To create an antireflective, superhydrophobic (i.e., *θ*_s_ > 150°) surface, we propose a hierarchical design, in which close‐packed, deep‐subwavelength‐scale (20 nm in diameter) colloidal silica (CS) NPs and submicron‐length, chain‐like fumed silica (FS) NPs are coated in sequence (Figure 1b). The lower CS NPs and the upper FS NPs serve as a broadband (400–800 nm) antireflective layer and a superhydrophobic self‐cleaning layer, respectively. Such multifunctional surfaces were obtained by iterating facile spraying processes for CS and FS NPs (**Figure** **2**a,b, and the Experimental Section). The key challenges in coating CS NPs involve how to assemble them with a constant packing density and to precisely adjust their total thickness to the quarter‐wave condition across the entire substrate area, both of which are pertinent to the antireflection capability. To this end, we prepared positively charged glass substrates by immersing them in a cationic poly(diallyldimethylammonium chloride) (PDDA) solution, such that negatively charged CS NPs uniformly adhered to the substrate during the spray coating process (Experimental Section).^[^ ^18^ ^]^ Then, CS NPs were vertically stacked in a face‐centered cubic (FCC) lattice with maximum packing density during the spraying process (Figure 2c,d) because the lateral array of CS NPs clung to the substrate established a well‐organized base plane. This feature is important because the 3D packing of CS NPs can be treated as a homogeneous layer with a low effective index, which was confirmed by conducting spectroscopic ellipsometry measurements on fabricated CS NPs (Figure 2e), thereby providing a haze‐free, antireflective surface. We have provided cross‐sectional transmission electron microscopy (TEM) images of our spray‐coated CS NPs to demonstrate their 3D close packing (Figure S1, Supporting Information).

A superhydrophobic surface can be obtained only if the area of the hydrophobic surface is augmented via a roughening process. The *r*_q_ and *θ*_s_ of fabricated close‐packed CS NPs were 9.6 nm and 35°, respectively, which were similar to those of bare glass (i.e., 3.0 nm and 21°) with a hydrophilic surface (Figure 2b). However, the additional coating of FS NPs, which partly covered the top surface of the CS NPs, dramatically increased the *r*_q_ of 98.7 nm. As silica NPs are initially hydrophilic because of the presence of silanol (Si‐OH) groups on their surfaces, we applied a postsurface treatment using a 1H,1H,2H,2H‐perfluorodecyltrimethoxysilane (HTMS) solution to render hydrophobicity (Experimental Section). The introduced FS/CS NP coating exhibited *θ*_s_ of 170° ± 3.3°, which was produced by a combination of the surface morphology and low surface energy (HTMS) treatment based on Cassie–Baxter equation.^[^ ^30^ ^]^

Notably, the hydrophobically treated FS/CS NP surface exhibited *θ*_sa_ < 1°, thereby enabling a self‐cleaning function on nearly planar devices (Movie S1 and Figure S2, Supporting Information). The near‐zero *θ*_sa_ indicates that our coating can substantially reduce the contact line depinning force and facilitate the droplet removal, which is unique to our hierarchical design.

Figure 2c shows the camera and scanning electron microscopy (SEM) images obtained at each fabrication step illustrated in Figure 2a. For bare glass, a significant amount of reflection limits the visibility of the background at an oblique viewing angle under sunlight. In comparison, the CS NP‐coated glass exhibited an enhanced level of transparency, thus displaying a high‐resolution (i.e., haze‐free) background without blurring.^[^ ^38^ ^]^ More importantly, this feature remains unchanged for a FS/CS NP‐coated sample. The FS NPs form an “island‐like morphology” and are present in patches, as evident from the SEM images in Figure 2c (rightmost). Taken together, while the additional coating of FS NPs does not affect the anti‐reflection capability of the CS NP coating, it creates a superhydrophobic surface because of the increased *r*_q_ value.

As previously mentioned, precise control over the coating thickness (*t*) of the CS NPs dictates the antireflection capability of the hierarchically designed surfaces. In this spraying process, the use of CS NPs with different concentrations in an aqueous solution yielded *t* values over one optical cycle using a fixed spraying time (Figure 2d,f, and the Experimental Section). The cross‐sectional SEM images of CS NP coatings (labeled I to V in Figure 2d) indicate that they possess a close‐packed density, irrespective of the concentration of CS NPs. Moreover, *t* values retain a linearity with respect to the concentration of CS NPs (Figure 2f); they monotonically shift from 48 nm (0.3 wt%) to 123 nm (1.1 wt%). These results verify the advantages of the spraying process to achieve antireflective superhydrophobic surfaces.

### 2.2 Assessment of Antireflection and Haze‐Free Performances

**Figure 3:**
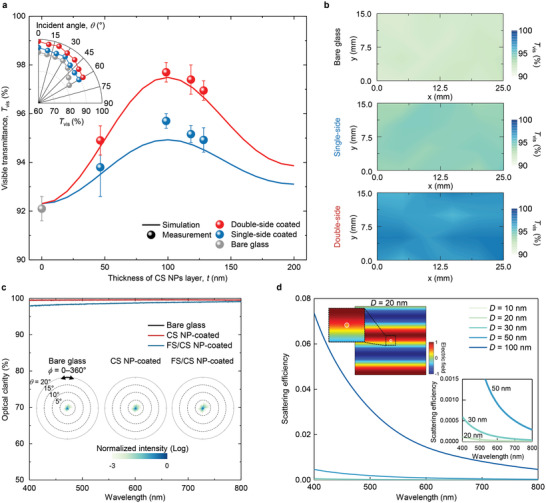
Assessment of antireflection and haze‐free performance. a) Measured (symbol) and simulated (solid line) average visible transmittance (*T*_vis_) for single‐ and double‐side CS NP‐coated glass with various *t* values. Each error bar represents the standard deviation from three independent measurements at three distinct positions on three separate samples. Insets: measured angle‐resolved *T*_vis_ for the bare and single‐ and double‐side CS NP‐coated samples. b) Measured position‐dependent *T*_vis_ values for the bare and single‐ and double‐side CS NP‐coated samples. c) Measured wavelength‐resolved optical clarity spectra for the bare, CS NP‐, and FS/CS NP‐coated samples. Insets: measured wavelength integrated far‐field transmission profiles for the bare, CS NP‐, and FS/CS NP‐coated samples, respectively. d) Scattering efficiencies of single‐side NP coated glass with *D* of 10, 20, 30, 50, and 100 nm. Inset: simulated scattered electric‐field profile for *D* = 20 nm, single‐NP, obtained at a wavelength of 530 nm.

We determined the visible transmittance values (*T*_vis_) of single‐ or double‐side CS NP‐coated glass with various *t* values by conducting optical measurements and simulations (**Figure** **3**a and the Experimental Section). Each *T*_vis_ was acquired by averaging the transmittance at each visible wavelength (380–780 nm), weighted by the AM 1.5G solar spectrum (Experimental Section). We fabricated four samples for each concentration of CS NPs and performed a statistical analysis to determine *T*_vis_. The measured results indicate that both the single‐ and double‐side coated samples maximize their *T*_vis_ values (95.7% and 97.7%, respectively) at *t* = 100 nm, corresponding to 0.7 wt% in Figure 2f, which surpass the 92.1% value for bare glass (symbols in Figure 3a). At *t* = 100 nm, the transmittance is maximized in the green wavelength region (495–570 nm), which is spectrally matched with the peak intensity of the solar spectrum (Figure S3, Supporting Information). To support these measured data, we performed rigorous coupled‐wave analysis (RCWA) simulations for various *t* values (0–200 nm) (lines in Figure 3a; and Figure S4, Supporting Information). The simulated results are well fitted to the sinusoidal functions that represent an antireflection feature. Note that for the RCWA simulations, the used effective index (*n*_eff_ ≈1.35) was derived from the spectroscopic ellipsometry measurements (Figure 2e), which is nearly the same as *n*_eff_ of CS NP with maximum packing density in the FCC lattice (Experimental Section). The good agreement between the measured (Figure 3a, symbols) and simulated (Figure 3a, lines) data suggest that the fabricated coatings using CS NPs possess a packing density value similar to that of the FCC lattice. Moreover, the *n*_eff_ of 1.35 is closer than the optimal refractive index (*n*) of antireflection coating used on glass.

For the samples where *t* = 100 nm, the antireflection performance remains effective over a large range of viewing angles (*θ*), as verified by angle‐resolved transmittance measurements (Figure 3a, inset). Both the single‐ and double‐side coated samples retained *T*_vis_ values >90% for *θ* < 60°, whereas bare glass exhibits a significant drop in *T*_vis_ values for *θ* > 45°. This omnidirectional feature is important for applications that require broad incident angles, such as displays. Furthermore, we obtained position‐dependent *T*_vis_ values across the substrate area to evaluate the uniformity of the coating thickness (Figure 3b; and Figure S5a, Supporting Information, and the Experimental Section). The heat maps confirm the antireflection performance of the CS NP‐coated samples and, more importantly, verify the homogeneity of our spray‐coating process at the wafer scale without the performance degradation by particle agglomeration.

Notably, the CS NP and FS/CS NP enhance the transparency of the coated bare glass without image degradation,^[^ ^38^ ^]^ which differentiates them from other antireflection techniques (Figures 1a and 2c). For quantitative analysis, we obtained the wavelength‐resolved optical clarity of the CS NP and FS/CS NP‐coated glass samples relative to bare glass (Figure 3c). Optical clarity is defined as the ratio of specular transmittance to total transmittance, which is acquired using an integrating sphere system (Figure S5b, Supporting Information; and the Experimental Section). The measured results reveal that both coatings using the CS and FS/CS NP almost preserve the optical clarity of bare glass in the visible wavelengths (Figure 3c). Quantitatively, the CS NP and FS/CS NP exhibit haze (i.e., using the standard ASTM D1003 method)^[^ ^41^ ^]^ values of 0.18% and 0.97% in the visible region, respectively, which does not significantly differ from that (≈0.17%) of bare glass (Figure S6, Supporting Information). These results are consistent with those of the optical clarity measurements and far‐field distribution, as shown in Figure 3c. This wavelength‐independent, high‐clarity feature was confirmed by obtaining far‐field distributions for normally incident light (Figure 3c, inset; and Figure S5c, Supporting Information; and the Experimental Section). Both the CS NP‐ and FS/CS NP‐coated samples exhibit a point‐like distribution, which is indicative of specular transmission, consistent with the results of the optical clarity measurements.

To support the measured results, we explored the scattering characteristics of the NPs using finite‐difference time‐domain (FDTD) simulations (Figure 3d).^[^ ^38^ ^]^ The scattering efficiencies of single NPs with diameter (*D*) of 10, 20, 30, 50, and 100 nm were calculated in the visible region (Experimental Section). The simulated results show that the scattering efficiencies exponentially decrease with decreasing *D* at all the considered wavelengths; they are essentially zero at *D* < 30 nm. For *D* = 20 nm, which is similar to the size of the CS NPs, a single NP marginally perturbs the wave front of an incident plane wave (Figure 3d, inset). These theoretical findings support the use of deep‐subwavelength‐scale NPs for attaining high‐clarity, antireflective coatings.

### 2.3 Assessment of Self‐Cleaning Performance

To create a superhydrophobic surface, an additional coating of FS NPs is essential in conjunction with a postsurface treatment. We prepared FS NP coatings with various concentrations (i.e., weight ratios of 0.1–0.4 wt% in a fumed silica‐based solution) to investigate the effects of different concentrations (Figure S7a, Supporting Information). The top‐view SEM images show that the covered area ratio of FS NPs gradually increases with increasing concentration (Figure S7b, Supporting Information), and reaches ≈0.45 at 0.3 wt%. Note that *θ*_s_ is typically influenced by many environmental factors such as the volume of a water droplet, temperature, humidity, and even fitting modes.^[^ ^40^ ^]^ Therefore, it is only used to roughly predict the wetting property of a given surface. As auxiliary metrics, we obtained dynamic contact angles (i.e., advancing (*θ*_a_) and receding (*θ*_r_) contact angles). Notably, *θ*_a_ and *θ*_r_ of the FS NP‐coated surfaces are constant, irrespective of the concentration (Figure S7c, Supporting Information). However, the transmittance of FS/CS NP‐coated samples slightly decreases at 0.4 wt% (Figure S8a, Supporting Information), we selected the 0.3 wt% concentration as the optimal FS NP concentration.

**Figure 4:**
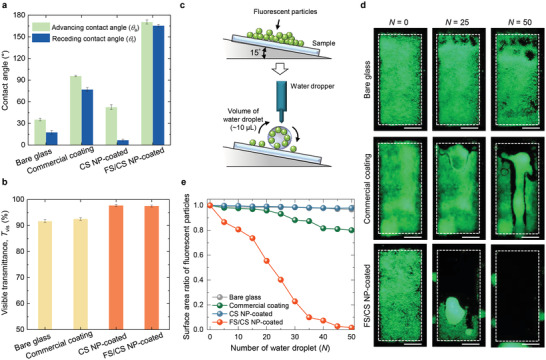
Assessment of self‐cleaning performance. a,b) Measured *θ*_a_ and *θ*_r_ a), and *T*_vis_ b) for the bare, commercial coating, CS NP‐, and FS/CS NP‐coated glass. Each error bar represents the standard deviation from three independent measurements at three distinct positions on three separate samples. c) Schematic of self‐cleaning test setup. d,e) Measurements of self‐cleaning capability in different samples where 200 µm diameter fluorescent particles were used as contaminants. Photo images d) obtained at *N* = 0, 25, and 50 for the bare, commercial coating and FS/CS NP‐coated glass. Scale bars: 1 cm. Surface area ratio e) of the remaining fluorescent particles at *N* water droplets to the bare, commercial coating, CS NP‐coated, and FS/CS NP‐coated glass.

We compared the *θ*_a_ and *θ*_r_ values of FS/CS NPs with those of bare glass, a commercial coating, and CS NP coating (**Figure** **4**a and the Experimental Section). The measured results show that the FS/CS NPs only induce a superhydrophobic surface with *θ*_a_ and *θ*_r_ > 165°. Contact angle hysteresis (*θ*_a_–*θ*_r_, *∆θ*) is determined by the energy barriers required for the movement of a droplet from one quasistatic state to another on a surface.^[^ ^40^ ^]^ According to the El Sherbini and Jacobi model,^[^ ^42^ ^]^ an adhesion force (*F*_adh_) between a droplet and a surface is given as *F*_adh_ = *kLγ*(cos*θ*_a_–cos*θ*_r_), where *k* is a geometry factor of the droplet, *L* is the size of the droplet and *γ* is the liquid–vapor surface tension. This equation indicates that if *∆θ* is close to zero, the droplet immediately slides on a surface without substantial friction.^[^ ^1^, ^4^, ^40^ ^]^ In addition, measurements on multiple samples show that our coating exhibits a *∆θ* of 5.1° ± 2.6° and *θ*_s_ of 170° ± 3.3°, which verifies its outstanding droplet mobility as compared with other previous implementations in Figure 1a. In comparison, the Δ*θ* (or *θ*_s_) value of only the CS NP coating after the surface treatment are much higher (or lower) than those of the FS/CS NP coating (Table S2, Supporting Information). These results indicate that although only the CS NP coating yields a haze‐free antireflection, it does not satisfy the criterion for superhydrophobicity because of its low *r*_q_ (i.e., 9.6 nm), as shown in Figure 2b.

Moreover, the FS/CS NP‐coated glass preserves the ultrahigh transmittance (97.5%) of the CS NP‐coated glass, exhibiting enhancements of 6% and 5% relative to the bare glass and commercial coating, respectively (Figure 4b). To elucidate how the additional coating using FS NPs does not deteriorate the transmittance, we performed RCWA simulations to evaluate changes in transmittance (Δ*T*) at various areas coated by FS NPs (Figure S8b,c, Supporting Information). The simulated results indicate that Δ*T* is <1% when the covered area ratio is <0.5, which is consistent with the measured results; the covered area of the 0.3 wt% FS NP‐coated sample is 0.45, as shown in Figure S7a,b, Supporting Information. Control experiments on only the FS NP coatings with various covered area ratios (i.e., concentrations of FS NPs) are necessary to justify our hierarchical design consisting of FS/CS NPs; “haze‐free” antireflection and self‐cleaning capabilities are only feasible by using the double‐layered FS/CS NP coatings (Figure S9, Supporting Information). The control experiments show that the transmittance is nearly equal to that of bare glass for low covered area ratios and it is greatly reduced for high covered area ratios owing to the scattering of “aggregated” FS NPs (Figure S9a–d, Supporting Information). Consequently, only the FS NP coatings cannot lead to haze‐free antireflection, although it renders superhydrophobicity (Figure S9d,e, Supporting Information).

The self‐cleaning ability of outdoor surfaces is affected by numerous environmental and technical factors, such as weather, location, pollution level, geometry, and tilting angle. The purpose of the self‐cleaning tests in this work is to quantitatively assess how superhydrophobic coatings with extremely low hysteresis benefit the self‐cleaning phenomenon under controlled setting. The concept of the self‐cleaning test was shown in Figure 4c. For these experiments, single water droplets (≈10 µL in volume) fall onto a sample at constant time intervals and roll down a surface with an inclination angle of 15° (Experimental Section). In previous studies, the majority of dust particles deposited on PV modules in natural environments have sizes in the range of dozens of micrometers.^[^ ^43^, ^44^, ^45^ ^]^ Dust particles with a diameter of up to 400 µm are observed.^[^ ^46^ ^]^ Two types of fluorescent particles with different sizes (200 or 30 µm on average) were used to investigate the self‐cleaning capability in accordance with the previously reported particle sizes (Figure S10a, Supporting Information). Similar‐sized particles were used in a number of self‐cleaning studies, including sea sand with a diameter of 100–300 µm,^[^ ^43^ ^]^ carbon nanotube with a diameter of 10 µm,^[^ ^47^ ^]^ and fly‐ash with a diameter of 45 µm,^[^ ^47^ ^]^ and red clay with 25 µm.^[^ ^48^ ^]^ Although small particles such as airborne PM2.5 particles can potentially be a significant source of contamination in a specific area, we focused on the self‐cleaning tests using particles with sizes of 30 and 200 µm because it is challenging to execute well‐controlled and quantitative experiments with such small particles. The fluorescent particles used in this study provide green fluorescence emission when exposed to a white light source (Experimental Section), which can increase contrast viewing before and after self‐cleaning experiments in dark lighting conditions. This property enables the distinction between the coverage of self‐cleaned and fluorescent particle‐remained areas. Therefore, the temporal change in the area of the remaining fluorescent particles is quantitatively identified as the self‐cleaning capability of the sample (Movies S2 for FS/CS NP coating and S3 for bare glass, Supporting information).

At the outset, a sample is completely covered with fluorescent particles. We obtained photo images under the exposure of a pumping light source at incremental numbers of water droplets (*N*) using bare glass, a commercial coating, and FS/CS NP (Figure 4d). Based on these images, we acquired the area in which fluorescent particles remained (Figure 4e). For these measurements, relatively large (200 µm) fluorescent particles were used. The FS/CS NP impart high mobility to a water droplet owing to their large *θ*_s_ and low *∆θ* values. Consequently, the water droplets continuously sweep the fluorescent particles away as they travel along the surface, in contrast to the other reference samples (Figure 4d). For the FS/CS NP‐coated surface, the area of the remaining fluorescent particles steadily decreased with increasing *N*; self‐cleaning was successfully completed when *N* < 50 (Figure 4e).

We conducted the same self‐cleaning experiments using relatively small (30 µm) fluorescent particles and observed the same behavior for the FS/CS NP‐coated samples (Figure S10b,c, Supporting Information). In comparison, for bare glass and CS NP‐coated glass, the swept area at *N* = 50 improves somewhat compared to the case using large fluorescent particles, although both images appear smudged across the entire sample area (Figure S10d, Supporting Information). Furthermore, we conducted the same self‐cleaning test with two types of fluorescent particles (200 and 30 µm on average in sizes) at an inclination angle of 8° (Figure S11, Supporting Information). For both fluorescent particles, the swept area steadily increased on the superhydrophobic surface with *N*. Subsequently, complete self‐cleaning was achieved at *N* ≈ 60; this saturation value was slightly larger than the result with an inclination angle of 15° (*N* ≈ 50), as shown in Figure 4e; and Figure S10b, Supporting Information. In addition, due to their relatively light weight, small particles (30 µm) tend to be removed more quickly than large particles (200 µm) in the investigated size range (Figure S11, Supporting Information). These experimental findings extend the application of our coating to outdoor displays and signs, horizontal, or slightly tilted windows, and automobiles with solar panel roofs.

We performed a quantitative comparison of our FS/CS coating with previously reported “transparent” hydrophobic coatings in terms of *T*_vis_ and *θ*_s_ values (Figure 1a). Most of the previous studies were based on top‐down^[^ ^6^, ^7^, ^8^, ^9^, ^10^, ^11^, ^12^, ^13^, ^14^ ^]^ or bottom‐up^[^ ^15^, ^16^, ^17^, ^18^, ^19^, ^20^, ^21^, ^22^, ^23^, ^24^, ^25^, ^26^, ^27^, ^28^, ^29^ ^]^ approaches and achieved decent transmittance. However, regarding both *T*_vis_ and *θ*_s_ metrics, our developed FS/CS NP coating significantly outperforms other coatings. In addition, considering the minimum usage of raw materials (≈1.0 wt% silica in a solution) and low‐temperature (<90 °C) surface treatment of the spraying process developed herein, the FS/CS NP coating provides an economically feasible means to implement a haze‐free broadband antireflection, self‐cleaning technology.

### 2.4 Photovoltaic Experiments and Reliability Tests

**Figure 5:**
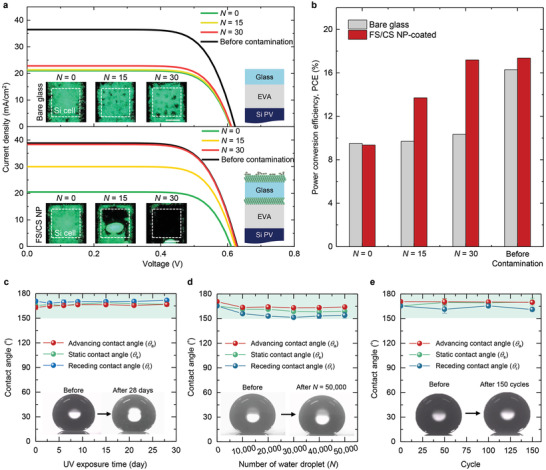
Photovoltaic experiments and reliability test. a) Measured *J–V* curves of PV device with bare (upper) and FS/CS NP‐coated glass (lower) samples at *N* = 0, 15, and 30, and before contamination, respectively. Insets: employing structures on PV device. Scale bars: 1 cm. b) PCE values of PV device with bare and FS/CS NP‐coated glass samples in (a). Inset: photo images obtained at *N* = 0, 15, and 30. Scale bar: 1 cm. c–e) Mechanical stability measurements of the FS/CS NP‐coated sample. The measured *θ*_a_, *θ*_r_, and *θ*_s_ values of the FS/CS NP coating after exposure of accelerated environmental stress coupled with UV irradiation and high ambient temperature environment c), after water droplet impacts on the sample d), and after repetitive thermal stress e), respectively. Insets: photo images showing that water droplets retain spherical shape after each reliable test.

Solar energy systems, including concentrated PVs, are mostly built in semiarid and arid regions to harness abundant sunlight. However, in such places, PV panels are heavily covered with dirt particles, which deteriorate their PCE and reliability over time. To address these issues, we adopted the FS/CS NP strategy to polycrystalline‐Si PV devices. Such opaque PV devices require only self‐cleaning and antireflection functions. However, we note that semitransparent PVs, which are gaining immense attention as a strategy for solar energy harvesting on buildings and automobiles, indeed require a haze‐free characteristic.^[^ ^36^ ^]^ For these self‐cleaning experiments, we obtained the current density–voltage (*J*–*V*) curves of PV devices encapsulated with ethylene vinyl acetate that were bonded to bare glass or to FS/CS NP‐coated glass (**Figure** **5**a; and Figure S12a, Supporting Information). Because the size distribution of dirt particles is typically within 30–160 µm,^[^ ^49^ ^]^ the same large (200 µm) and small (30 µm) fluorescent particles were used to emulate a dusty environment. For both types of fluorescent particles, the intact *J*–*V* curves of the FS/CS‐coated PV devices were rapidly recovered (*N* < 30), which is consistent with images captured at different *N* values (Figure 5a; and Figure S12a, insets, Supporting Information). Based on the measured *J*–*V* curves, we acquired the PCE values of the two types of PV devices at various *N* values (Figure 5b; and Figure S12b, Supporting Information). At the outset (i.e., *N* = 0), when the samples are completely covered with fluorescent particles, the PCE values are nearly equal to or less than half of the intact values for contaminant sizes of 200 and 30 µm, respectively. Notably, the PV devices with the FS/CS NP coating regained their intact PCE values at *N* = 30 (200 µm) or *N* = 20 (30 µm). In contrast, the PCE values improved by only 5% (200 µm) and 24% (30 µm) relative to those at *N* = 0 for the bare glass PV devices. Moreover, even before contamination, the current density values for the PV devices covered with the FS/CS NP‐coated and bare glasses were 38.8 and 36.5 mA cm^−2^, respectively, which is consistent with the transmittance results shown in Figure 4b. As a result, the FS/CS NP‐coated PV devices exhibited a 6.1% improvement in power conversion efficiency compared with the bare glass PV devices. A few attempts at developing transparent superhydrophobic coatings for PVs have been reported.^[^ ^11^, ^13^, ^24^, ^26^, ^35^ ^]^ For example, Torun et al. created a transparent superhydrophobic coating using fluorinated NPs by spraying process.^[^ ^26^ ^]^ However, the PCE of PV devices after the coating was adversely reduced by ≈1.3% because of the lack of precise control over the coating morphology. Recently, Li et al. demonstrated an effective defrosting capability on PV devices using boehmite nanostructures.^[^ ^35^ ^]^ Such coating preserved the pristine performance of PV devices before and after defrosting experiments, providing a PCE enhancement of <3% due to the same degree of enhancement in visible transmittance. Furthermore, the fabrication involved a vacuum process (i.e., sputtering), which could limit overall scalability. We highlight that our precisely designed antireflective superhydrophobic coatings can enhance the efficiency of optoelectronic devices and maintain their performance in a dusty environment by using a scalable and high‐precision spraying process.

The newly introduced FS/CS NP coating may be frequently exposed to environmental stress such as ultraviolet (UV) irradiation, drop impacts and thermal stress, when it is applied to outdoor displays and signs, horizontal or slightly tilted windows, and photovoltaic cells. Hence, we examined the reliability of the FS/CS NP coatings under exposure to UV irradiation (Figure 5c), to water droplets (Figure 5d) and to thermal stress (Figure 5e). For the UV test, a light source produces 10.3 mW cm^−2^ in the wavelength region of 280–360 nm, which is approximately five times greater than the UV intensity of one‐sun irradiation (Experimental Section). In addition, the samples were heated at 70 °C to accelerate the UV test. Under these harsh conditions, we observed a marginal change in *θ*_a_, *θ*_r_, and *θ*_s_ values over 28 days. Likewise, for the water droplet and thermal stress tests, the FS/CS NP coatings maintained the superhydrophobic state (*θ*_s_ > 150° and *∆θ* < 10°) after 50 000 droplets and 150 temperature cycles. Comparison of the atomic force microscope (AFM) and SEM images obtained before (Figure 2b,c) and after (Figure S13, Supporting Information) the reliability tests reveal that the FS/CS NP coatings almost maintain its pristine *r*_q_ values (Figure S13a–c, Supporting Information), which is indicative of a marginal change in *θ*_a_, *θ*_r_, and *θ*_s_ values as shown in Figure 5c–e.

These results demonstrate that the adhesion between the sprayed particles and substrate is adequate to be used in the intended applications such as outdoor displays, signs, windows, and photovoltaic cells. Although we did not make detailed investigations, the developed surfaces would be only partially resistant to mechanical wear. In our hypothesis, mechanical wear would have a more significant impact on the self‐cleaning performance as the top HTMS‐treated fumed silica layer is damaged. However, using more mechanically resistant coating materials can help with applications that ask for a higher level of mechanical durability.

## 3 Conclusions

In this study, we developed silica NPs‐based superhydrophobic self‐cleaning surfaces with haze‐free and antireflection optical characteristics. A scalable and high‐precision spraying process produced layer‐by‐layer coatings consisting of two types of silica NPs with distinct morphologies; their antireflection and self‐cleaning performances were uniform over the entire sample area. These NP‐coated surfaces yielded a visible light transmittance of 97% through glass with minimal light scattering. In addition, their superhydrophobicity featuring a record‐high contact angle of >170° and a contact angle hysteresis of <10° enabled complete self‐cleaning with respect to various sizes of contaminants, outperforming a commercial coating. These optical and wetting functionalities enhanced the power conversion efficiency of glass‐cover‐bonded Si PV devices by 6.1% and promptly regained pristine device performance after being covered with dense contaminants. The developed coating technology tolerated adverse environmental conditions, including prolonged exposure to strong UV light, high temperatures, and continuous rainfall, thus preventing performance degradation or loss of materials. Furthermore, defrosting experiments on the developed NP‐coated surface verify that they can still work for self‐cleaning in cold climates (Figure S14, Supporting Information). Moreover, such weather resistance and mechanical durability can be further improved by introducing a low‐surface‐energy passivation layer (e.g., polytetrafluoroethylene) with high mechanical, thermal, and chemical stability.^[^ ^50^ ^]^ We believe that these multifunctional optical surfaces can be leveraged to resolve the temporal deterioration of performance associated with solar‐thermal energy harnessing devices, switchable windows, optical filters, and imaging devices used in outdoor applications.

## 4 Experimental Section

### Fabrication of Hierarchical Surface

Commercial slide glass (LK Lab) was used as the bare glass substrate. First, the bare glass was cleaned with ethanol for 5 min in an ultrasonication bath. To prepare the solution for enhancement of adhesion between the negatively charged CS NPs and glass substrate, cationic poly(diallyldimethylammonium chloride) (PDDA) (20 wt%, Sigma‐Aldrich) was diluted to 0.1 wt% in an aqueous solution. Then, the sample was immersed in the diluted cationic PDDA solution for 15 min, followed by rinsing with deionized (DI) water and drying with N_2_ gas. The solution for the antireflective coating was prepared by mixing a CS NP solution (50 wt% in water, Ludox TM‐50, Sigma‐Aldrich), DI water, and ethanol. Different amounts of CS NP solution (from 0.1 to 1.1 g) were mixed in 15 g of DI water, and then 35 g of ethanol were added to the aqueous CS NP solution to reduce the surface tension of the coating solution. Concentrations of CS NPs in the coating solution were adjusted from 0.1 to 1.1 wt% using different amounts of CS NPs (from 0.1 to 1.1 g). Then, the coating solution was stirred at 200 rpm for 5 min. The aqueous solution was sprayed on the substrate at a distance of 10 cm from the nozzle (diameter: 0.7 mm) using a custom‐built spraying system. The nozzle speed, spraying pressure, and flow rate were maintained at 50 mm s^−1^, 50 kPa, and 1.5 mL min^−1^, respectively. After the spraying process, fabricated samples were dried in an oven at 70 °C. As the coating solution for the superhydrophobic layer, 0.1–0.4 g of FS NPs were added to ethanol. Concentrations of FS NPs were controlled from 0.1 to 0.4 wt% as the mass of FS NPs was varied from 0.1 to 0.4 g. After the FS NP coating solution was dispersed using a probe‐type ultrasonicator (750 W, 20 kHz) for 10 min, the FS NP coating solution was sprayed onto the substrate using the same procedure as for the CS NP coating. To achieve superhydrophobicity, the FS/CS NP coating was treated with 1H,1H,2H,2H‐perfluorodecyltrimethoxysilane (HTMS, Sigma‐Aldrich) solution through a chemical vapor deposition process at atmospheric pressure for 2 h in a tightly sealed container maintained at 90 °C. A sealed container was used to prevent excessive diffusion of HTMS.

### Surface Characterization

The measured *θ*_s_ values were obtained by using a droplet analyzer based on the Bashforth–Adams equation (SDS‐TEZD, FEMTOFAB).^[^ ^51^ ^]^ The commercial droplet analyzer provided the profile of a droplet, thus yielding *θ*_s_ by fitting the acquired profile to the Bashforth–Adams equation.^[^ ^51^ ^]^ For statistical analysis, the measurements were performed at three different positions for the three samples. The volume of water droplets used for measurements of *θ*_s_ and *θ*_sa_ were 6.3 and 11.4 µL, respectively. In particular, *θ*_s_ was obtained by varying the volume of water droplets between 5 and 11 µL. Measured *θ*_s_ (≈170°) was marginally altered in the considered volume range. Dynamic contact angles (i.e., *θ*_a_ and *θ*_r_) were obtained by increasing or decreasing the volume of a droplet. For the measurement of *θ*_a_, a water droplet of 5 µL was initially held on the surface, and water (5–10 µL) was injected through a syringe needle until the baseline (i.e., the point at which the water droplet contacts the surface) eventually advanced the surface. The measurement of *θ*_r_ was performed in the opposite way, where water was removed through the syringe needle from an initial water droplet of 15 µL until the baseline eventually receded the surface.^[^ ^52^ ^]^ AFM measurements were performed using a Park XE‐100 AFM instrument under ambient conditions. All images were captured in tapping mode with a typical resonant frequency of 250–300 kHz. Top‐view and cross‐sectional SEM images were obtained using a Carl Zeiss MERIN microscope at an imaging voltage of 10 kV.

### Optical Characterization

The transmittance of the prepared samples was characterized using a custom‐built measurement system. For the transmittance of normal or variable incident angles, a visible‐to‐near‐infrared spectrophotometer (Maya 2000 Pro, Ocean Optics), an integrated sphere (CSRM‐RTC‐060‐SL, Labsphere), and a high‐power (1000 W) xenon lamp (#66 921, Newport) were used (Figure S5a, Supporting Information). For the position‐dependent average transmittance, a programmed motor *x*–*y* scanning system coupled with a transmittance measurement setup was used to characterize the uniformity of the average transmittance of the prepared samples. Here, the maximum scanning area was 25.0 × 15.0 mm^2^ (Figure S5a, Supporting Information). The visible transmittance, *T*_vis_ was obtained by integrating the absorptivity, *t*(*λ*), at different incident angles and wavelengths, weighted by the AM 1.5G spectrum (*I*_AM1.5G_(*λ*)) over visible wavelengths ranging from 0.38 to 0.78 µm,

(1) $$T_{\mathrm{vis}}=\frac{\int_{0.38}^{0.78}t\left(\lambda \right)I_{\mathrm{AM}1.5G}\left(\lambda \right)d\lambda }{\int_{0.38}^{0.78}I_{\mathrm{AM}1.5G}\left(\lambda \right)d\lambda }$$

The optical clarity spectra were characterized using a custom‐built transmittance setup (Figure S5b, Supporting Information). For the measurement, a high‐Lambertian reflectance standard sample (SRS‐99‐020, Labsphere) was used as a reference. The optical clarity is expressed as

(2) $$\mathrm{clar}\mathrm{ity}=1-\left[\frac{T_{3}\left(\lambda \right)/T_{1}\left(\lambda \right)}{T_{2}\left(\lambda \right)}\right]$$

where “*T*_1_” represents the total transmittance with the reflectance standard in position and no sample. “*T*_2_” represents the transmittance spectra of the samples, and “*T*_3_” represents the diffused transmittance with the sample in position after the standard is removed.

The far‐field distributions of normally transmitted light were measured using a custom‐built far‐field scanner coupled with a spectrometer (USB4000, Ocean Optics) and a high‐power (1000 W) xenon lamp (#66 921, Newport) (Figure S5c, Supporting Information). The detector scanner was programmed to rotate along the polar (*φ*) and azimuthal (*θ*) directions with a step size of 1°.

### Optical Simulations

The commercial software package RCWA (DiffractMOD, Rsoft) was used to characterize the transmittance of the close‐packed CS NP layer and the effective index layer of the coated glass. For the deep subwavelength‐sized nanoparticles of the close‐packed CS NPs layer, the spatial resolution along the *z*‐direction was fixed at 0.5 nm and the harmonics value was 20. For the effective index of the layer‐coated glass, the spatial resolution along the *z*‐direction was 1 nm and the zero‐harmonics state existed (owing to the symmetry in the *x* and *y* directions). The applied effective index (*n*_eff_) was obtained by using spectroscopic ellipsometry (Elli‐SEU, Ellipso Technology). FDTD simulations were used to obtain the scattering efficiencies of single NPs with diameter (*D*) of 10, 20, 30, 40, and 100 nm in the visible wavelengths. The scattered field distributions were first obtained. Then, the scattering efficiency was determined by dividing the scattering cross section by the projected value of a single NP. For the simulations, a spatial resolution of 0.05 nm was used for the *x*‐, *y*‐, and *z*‐directions, and a perfectly matched layer condition was adopted along all terminated planes.

### Self‐Cleaning Tests

The self‐cleaning capability of prepared samples was investigated through the extracted coverage of contaminants on the samples with different dripping droplets.^[^ ^53^ ^]^ Two types of insoluble europium‐based fluorescent particles (alkaline earth metal aluminate oxide doped with europium, absorption wavelength: 300–450 nm) with different sizes (200 and 30 µm on average) were utilized as contaminants (Figure S10a, Supporting Information). The samples were first positioned with an inclination angle of either 15° or 8°, and then uniformly covered with fluorescent particles (initial cover density: 0.03 g cm^−2^). Using a white light source (LED light, 75 W), the fluorescent particles emitted green light in dark lighting conditions. Single droplets of 10 µL were dripped over the inclined samples at regular intervals while tests were being recorded by a CCD camera for quantitative analysis. The fluorescent images were captured using the test movies as *N* increased for each sample (Movies S2 for FS/CS NP coating and S3 for bare glass, Supporting Information). Then, an image analysis software was applied to determine the surface coverage area ratios of fluorescence particles as a function of *N*.

### Photovoltaic Experiments and Reliability Tests

The photovoltaic performance of crystalline‐Si PV devices was evaluated in terms of current density–voltage (*J*–*V*) characteristics using a solar simulator (PEC‐L01, Peccell) and source meter (2450, Keithley Instruments) under AM 1.5G and 1‐sun (100 mW cm^−2^) conditions. The 1‐sun intensity was calibrated using a power meter (1918‐R, Newport). The active area of a Si cell was 4 cm^2^. Scanning was carried out over a −0.1 to 1.2 V range at a rate of 0.27 V s^−1^ with a dwell time of 50 ms at each point. The test samples were prepared after dripping droplets onto 15° inclined contaminated samples. An accelerated environment test was conducted in a custom‐built UV chamber consisting of three 8 W UV‐B lamps with a wavelength of 280–360 nm (intensity: ≈10 mW cm^−2^) and a temperature control system (controlled temperature: 70 °C). The distance between the sample and the UV source was 10 cm. For the water droplet tests, water droplets (≈10 µL in volume) were continuously dripped onto a sample from a height of 30 cm and the sample was inclined at 40°. The thermal stress test was performed under an environment with a temperature of 20 °C and a humidity of 40 ± 5%. Then, a temperature of sample was controlled by a heating stage (FTIR600, LINKAM). The temporal rate of temperature increment/decrement was set to be 1.0 ± 0.4 °C s^−1^ during 150 cycles. The defrosting experiment was performed under an environment with a temperature of 18.0 °C and a humidity of 80 ± 5%. First, the initial temperature of the samples was −14.0 °C, and the frosting duration time was 2400 s using a Peltier cooling module (CP‐200, TE Technology), which was connected to a temperature controller (TC‐720, TE Technology) and a power supply (PS‐24‐25, TE Technology). After then, the samples were monotonically heated above 0 °C for 1800 s.

### Statistical Analysis

The data were expressed as a mean standard deviation (SD). Measurements taken at three distinct points on three separate samples were used to determine the coating thickness. Three independent measurements were performed at three distinct points on three different samples to determine the wettability of fabricated samples. Three independent measurements taken at three different points on three separate samples yielded the optical characteristics. The transmittance of fabricated coatings was calculated using RCWA (DiffractMOD, Rsoft) software. The covered area ratio of fluorescent particles was obtained using Matlab software for the quantitative examination of self‐cleaning capabilities.

## Conflict of Interest

The authors declare no conflict of interest.

## Supporting information

Supporting Information

Supplemental Movie 1

Supplemental Movie 2

Supplemental Movie 3

## Data Availability

The data that support the findings of this study are available from the corresponding author upon reasonable request.
