# Supplementary material for: A Scalable Haze‐Free Antireflective Hierarchical Surface with Self‐Cleaning Capability
Source: Adv Sci (Weinh). 2022 Jul 28;9(27):2202781. doi: 10.1002/advs.202202781 (PMC9507353; doi:10.1002/advs.202202781)
Supplement: Supplementary file 1 — Supporting Information [file ADVS-9-2202781-s003.pdf]

ADVANCED SCIENCE
Open Access

Supporting Information

for *Adv. Sci.*, DOI 10.1002/advs.202202781

A Scalable Haze-Free Antireflective Hierarchical Surface with Self-Cleaning Capability

Seungtae Oh, Jin-Woo Cho, Jihun Lee, Jeonghoon Han, Sun-Kyung Kim and Youngsuk Nam**

Supporting Information

A scalable haze-free anti-reflective hierarchical surface with self-cleaning capability

Seungtae Oh, Jin-Woo Cho, Jihun Lee, Jeonghoon Han, Sun-Kyung Kim, and Youngsuk Nam**

*sunkim@khu.ac.kr and ysnam1@kaist.ac.kr

Contents

1. Cross-sectional TEM images of close-packed CS NPs (Figure S1)
2. Sliding angle of the FS/CS NPs coated surface (Figure S2)
3. Transmittance spectra of single- and double-side CS NP-coated glass (Figure S3)
4. RCWA simulations for close packed CS NPs and effective index layer of coated glass (Figure S4)
5. Optical characteristic measurement setup (Figure S5)
6. Haze spectra measurements (Figure S6)
7. Dynamic contact angles of FS/CS NP coating at various covered area ratios of FS NP coating (Figure S7)
8. Optical properties of FS/CS NP coatings at various covered area ratios of FS NP coating (Figure S8)
9. Optical and wetting properties for only FS NP coatings with various covered area ratios (Figure S9)
10. Self-cleaning capability in small-sized contaminants (Figure S10)
11. Measurements of self-cleaning capability at a tilted angle of 8° (Figure S11)
12. Photovoltaic experiments for small-sized contaminations (Figure S12)
13. AFM and SEM images after reliability tests (Figure S13)
14. Defrosting experiments (Figure S14)
15. Previously reported transparent, superhydrophobic surfaces (Table S1)
16. Contact angles of hydrophobically-treated CS NP and FS/CS NP coatings (Table S2)

1. Cross-sectional TEM images of close-packed CS NPs

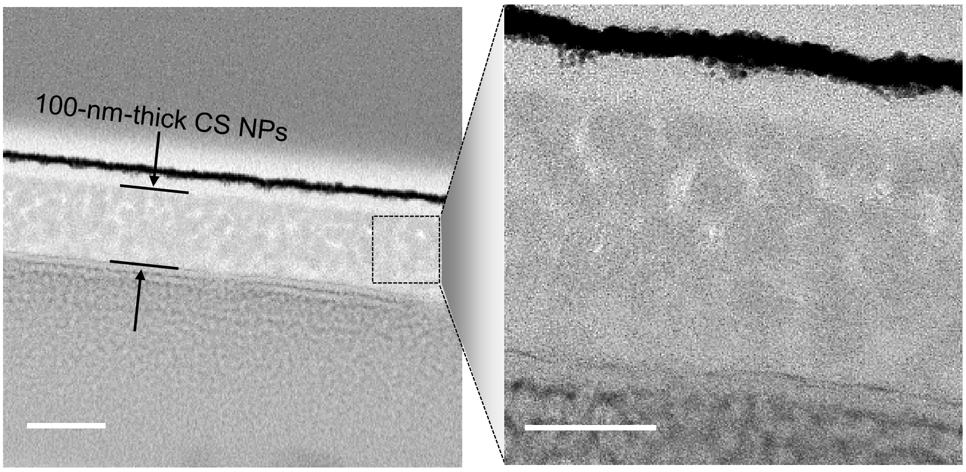

Figure S1. Cross-sectional TEM images of close-packed CS NPs. Scale bars: 100 nm (left) and 50 nm (right), respectively.

2. Sliding angle of the FS/CS NP coated surface

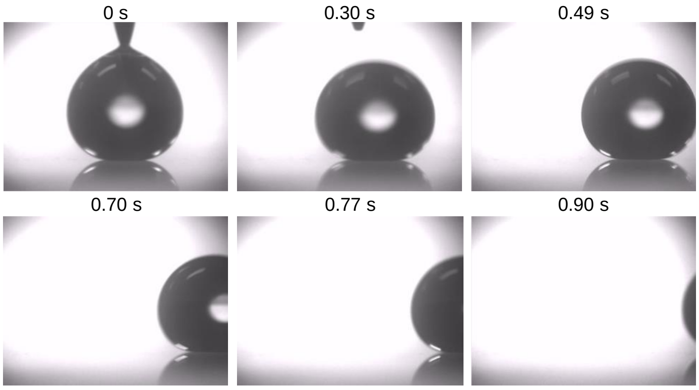

Figure S2. Sliding angle of the FS/CS NP-coated surface. Snapshots of a water droplet captured for 1 s when it hits a nearly planar (an inclination angle < 1°) surface with our developed FS/CS NP coatings.

3. Transmittance spectra of single- and double-side CS NP-coated glass

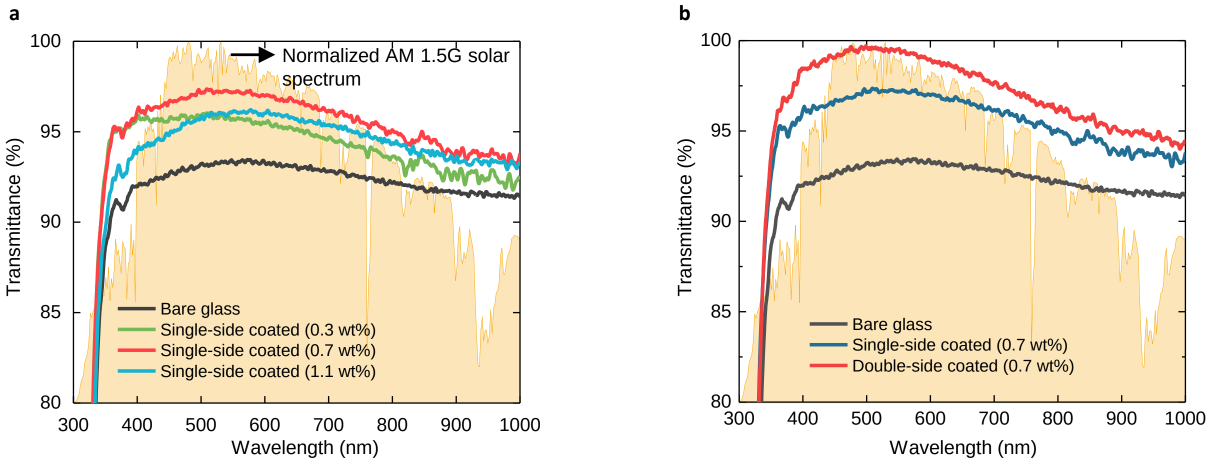

Figure S3. Transmittance spectra of single- and double-side CS NP-coated glass. a) Measured transmittance spectra of bare glass and single-side CS NP-coated glass with different concentrations (0.3, 0.7, and 1.1 wt%). b) Measured transmittance spectra of bare glass, single-, and double-side CS NP (0.7 wt%) coated glasses, respectively. For (a) and (b), the normalized AM 1.5G solar irradiance spectrum (yellow shaded area) is shown.

4. RCWA simulations for close packed CS NPs and effective index layer of coated glass

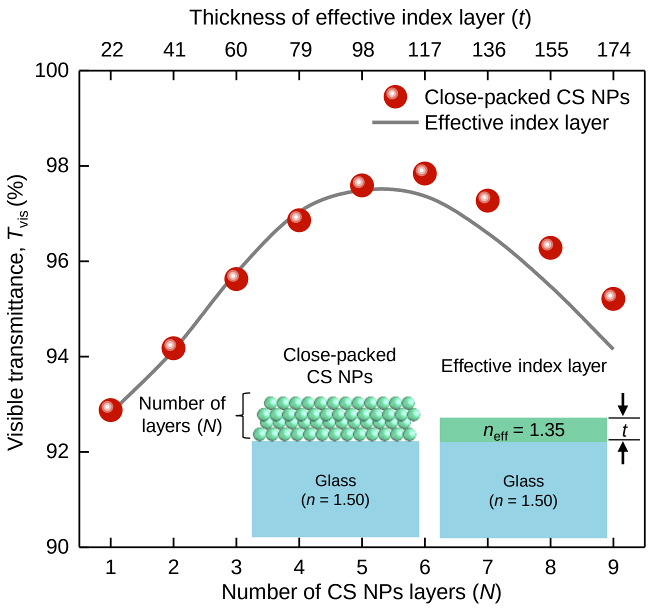

Figure S4. RCWA simulations for close packed CS NPs and effective index layer of coated glass. The values of T_{vis} corresponding to the close-packed CS NPs (symbols) and effective index layer (line), depending on the number of packed layers of CS NPs (*N*) and the thickness of the effective index film (*t*), respectively. Insets: schematics illustrating each simulated structure.

5. Optical characteristic measurement setup

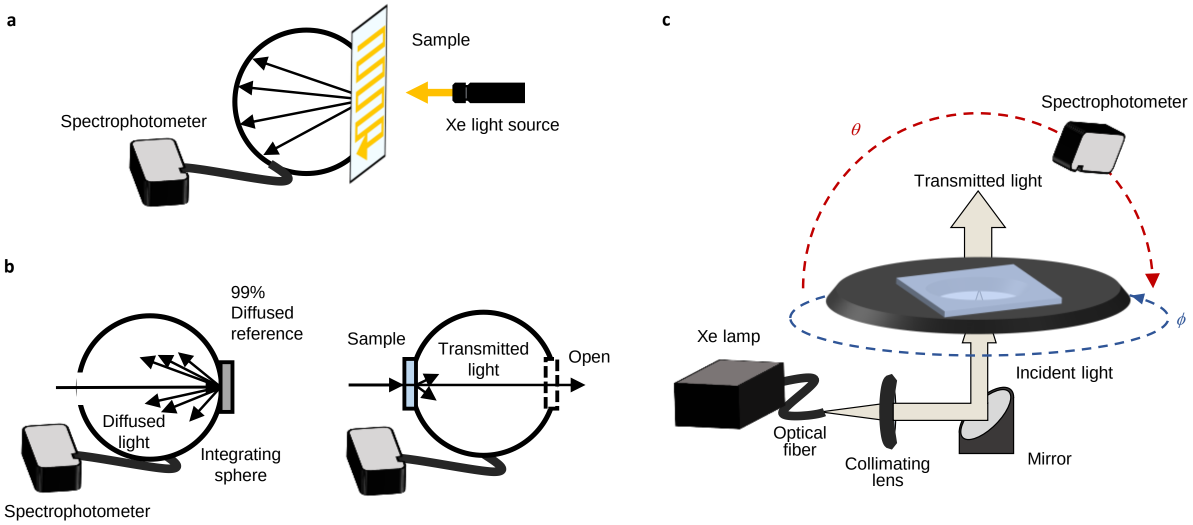

Figure S5. Optical characteristic measurement setup. a) Schematic illustrating a position-dependent T_{vis} measurement setup. b) Measurement setup for the optical clarity using a spectrometer with an integrating sphere. Schematic illustrating measurement setup for total transmittance (left) and diffused light after transmitting a sample (right). c) Schematic for measurement setup of the far-field transmission distribution for a normally incident light.

6. Haze spectra measurements

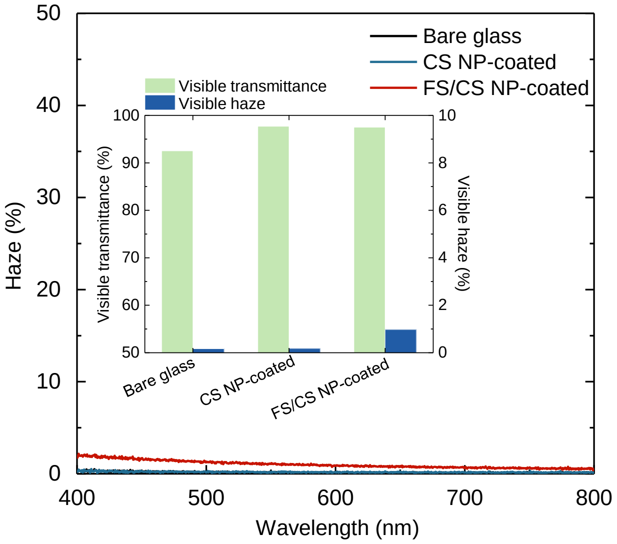

Figure S6. Haze spectra measurements. Measured wavelength-resolved haze spectra for the bare, CS NP-, and FS/CS NP-coated glasses. Insets: visible transmittance (T_{vis}) and visible haze values for the same samples.

7. Dynamic contact angles of FS/CS NP coating at various covered area ratios of FS NP coating

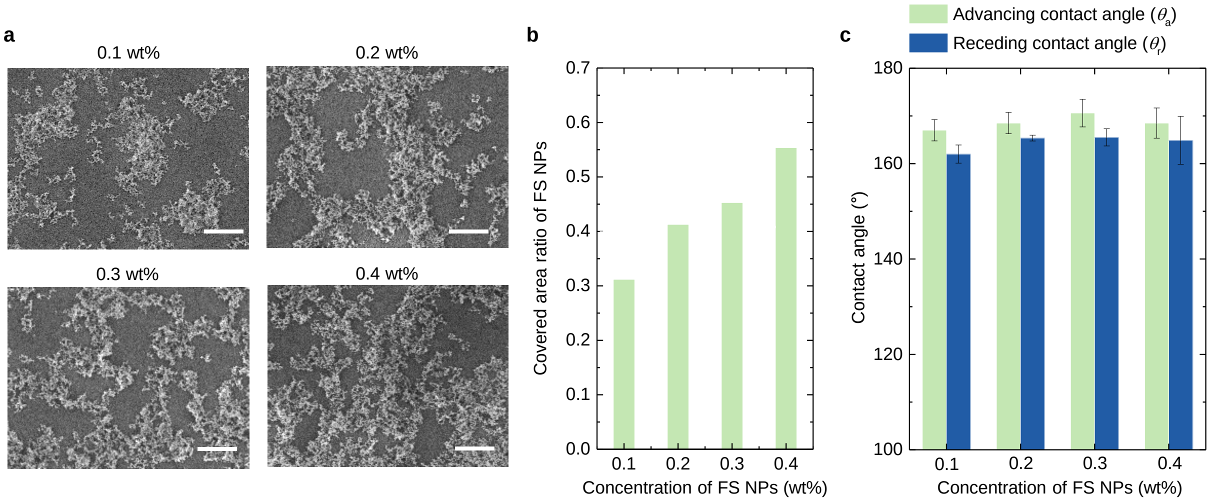

Figure S7. a) SEM images and b) covered area ratio of the FS/CS NP at various concentrations of FS coatings (e.g., 0.1, 0.2, 0.3, and 0.4 wt%). Scale bars: 1 μm. θ_a and θ_r values for the FS/CS NP coatings at various concentrations (e.g., 0.1, 0.2, 0.3, and 0.4 wt%).

8. Optical properties of FS/CS NP coatings at various covered area ratios of FS NP coating

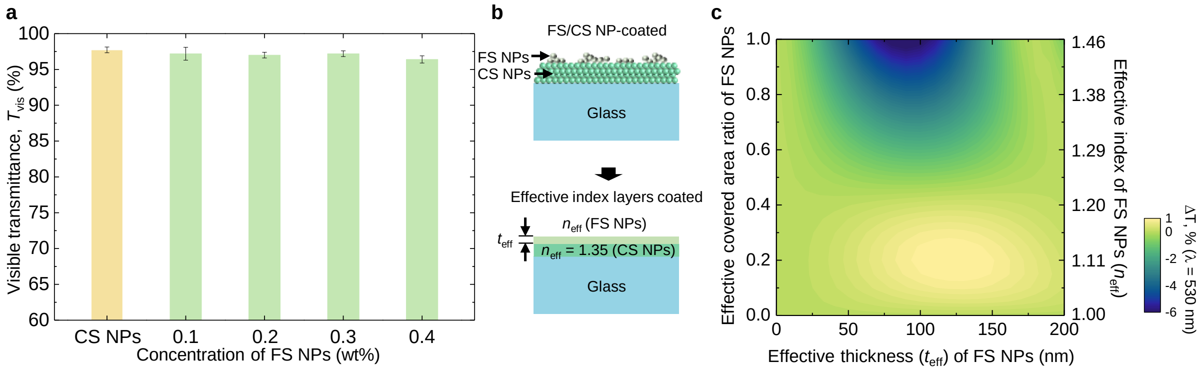

Figure S8. Optical properties of FS/CS NP coatings at various covered area ratios of FS NP coating. a) T_{vis} values for the FS/CS NP coatings at various concentrations of FS coatings (e.g., 0.1, 0.2, 0.3, and 0.4 wt%) and CS NP coatings as the reference in (a). b,c) Simulated effective index layer model for FS/CS NP-coated glass is represented in (b) and a surface plot represents ΔT (530 nm) in various surface areas of the FS NP coatings (c). Each error bar represents the standard deviation based on three independent measurements.

9. Optical and wetting properties for only FS NP coatings with various covered area ratios

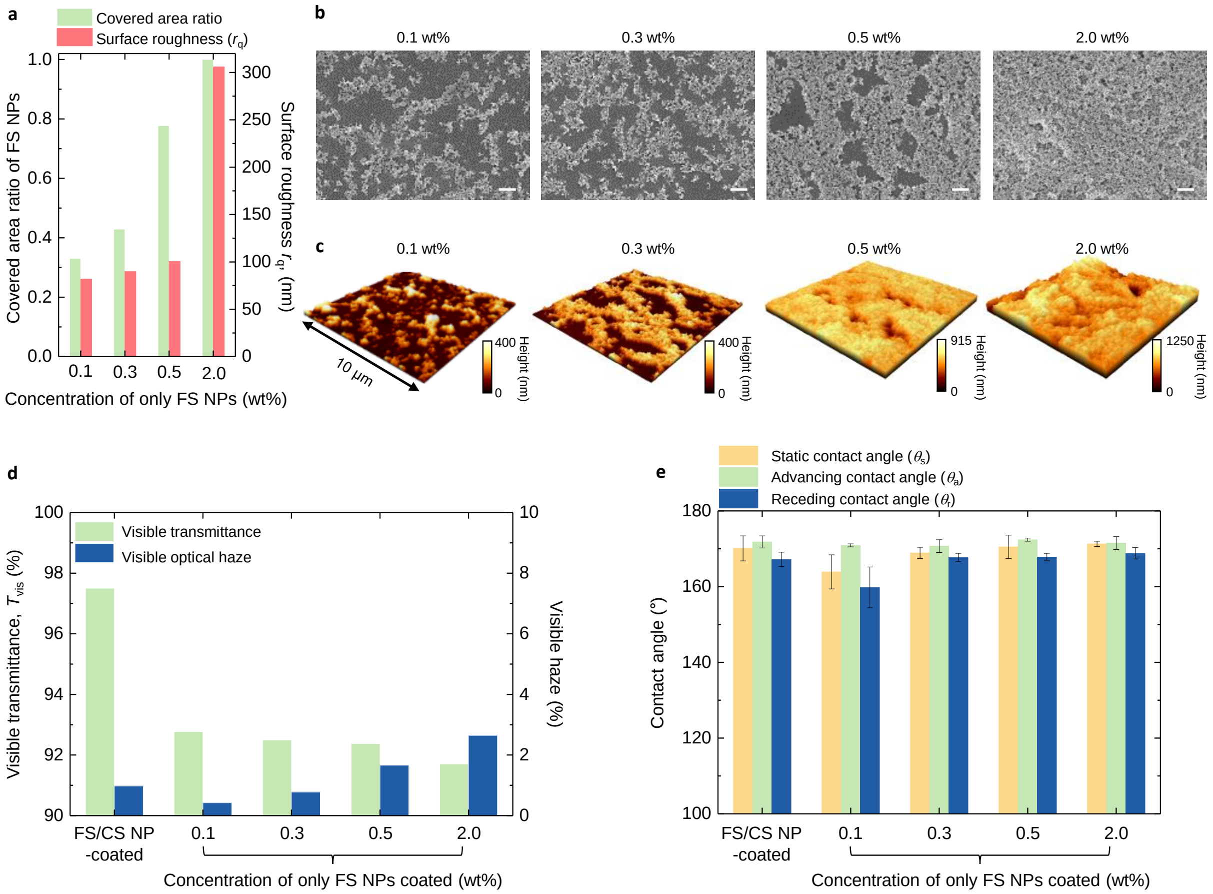

Figure S9. Optical and wetting properties for only FS NP coatings with various covered area ratios. a–c) Covered area ratio and r_q values (a), SEM (b), and AFM (c) images of the only FS NP coatings with various concentrations (e.g., 0.1, 0.3, 0.5, and 2.0 wt%). Scale bars: 1 μm. d,e) T_{vis} and haze values (d) and θ_s, θ_a, and θ_r values (e) of the FS/CS NP coating and the only FS NP coatings with various concentrations (e.g., 0.1, 0.3, 0.5, and 2.0 wt%).

10. Self-cleaning capability in small-sized contaminants

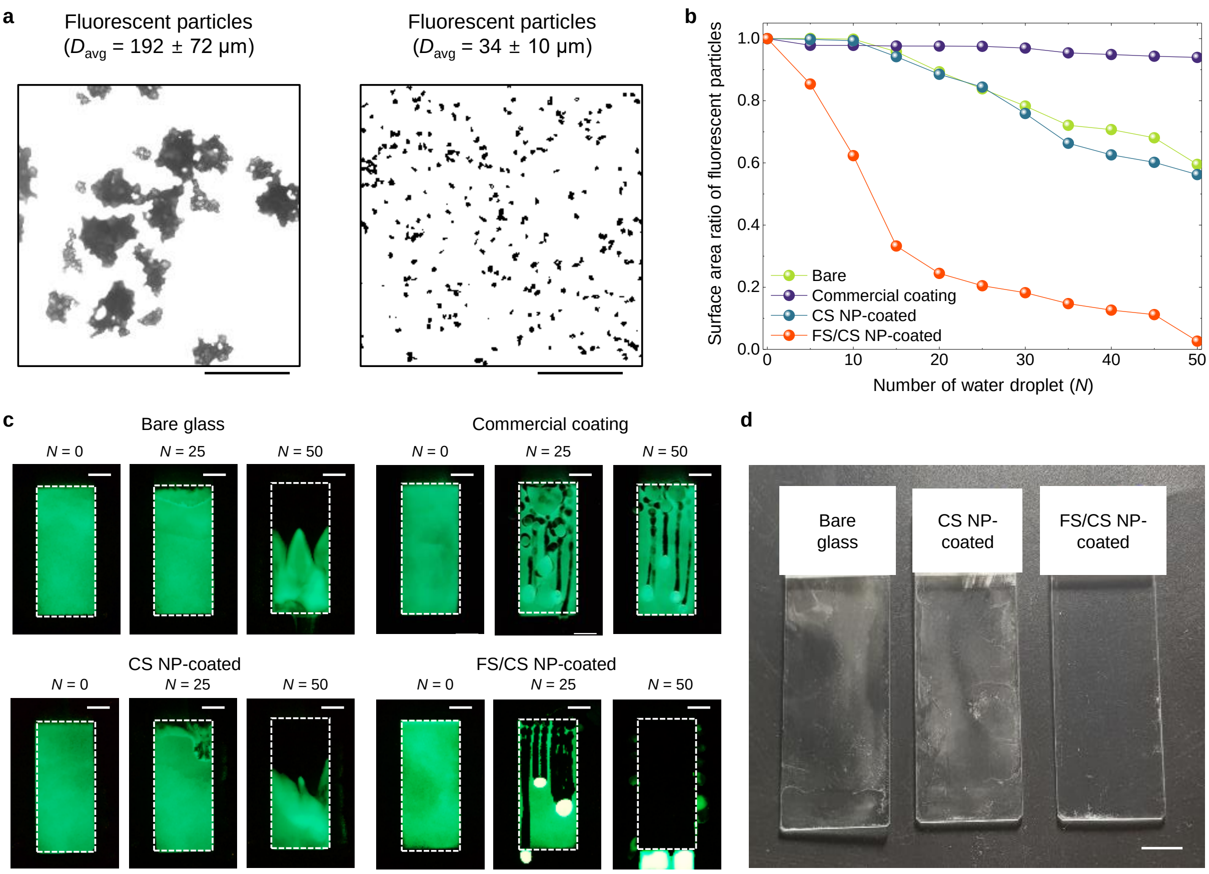

Figure S10. Self-cleaning capability in small-sized contaminants. a) Microscopic images of fluorescent particles for 200- (left) and 30-μm-diameter (right), respectively. Scale bars: 1 mm. b,c) Self-cleaning capability for 30-μm-diameter case. Surface area ratio (b) of the remaining fluorescent particles for *N* water droplets for bare glass, commercial coating glass, CS NP-, and FS/CS NP-coated glass. Photo images (c) are obtained for *N* water droplets on the four types of coated glass. Scale bars: 1 cm. d) Images obtained for *N* = 50 for bare, CS NP-, and FS/CS NP-coated glass samples after self-cleaning test using 30-μm-diameter fluorescent particles. Scale bar: 1 cm.

11. Measurements of self-cleaning capability at a tilted angle of 8°

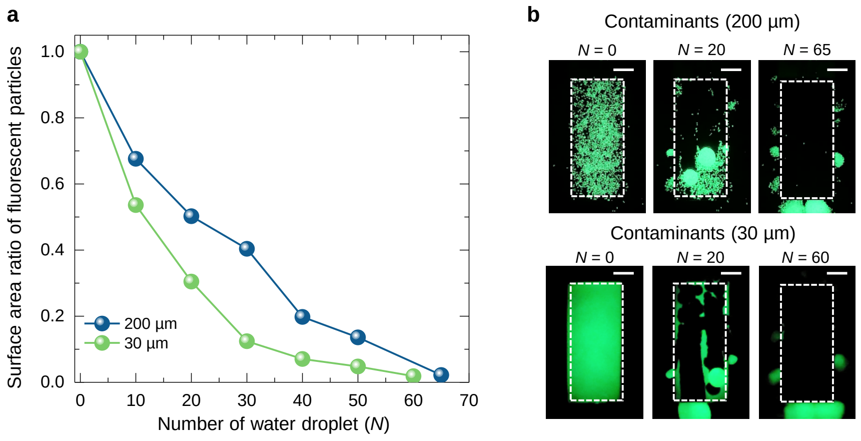

Figure S11. Measurements of self-cleaning capability at a tilted angle of 8°. a) Surface area ratio of the remaining fluorescent particles at *N* water droplets where 200- or 30-µm-diameter fluorescent particles were used as contaminants. b) Photo images obtained at *N* = 0, 20, and 65 (upper) and at *N* = 0, 20, and 60 (lower) where the 200- or 30-µm-diameter fluorescent particles were used as contaminants, respectively. Scale bars: 1 cm.

12. Photovoltaic experiments for small-sized contaminations

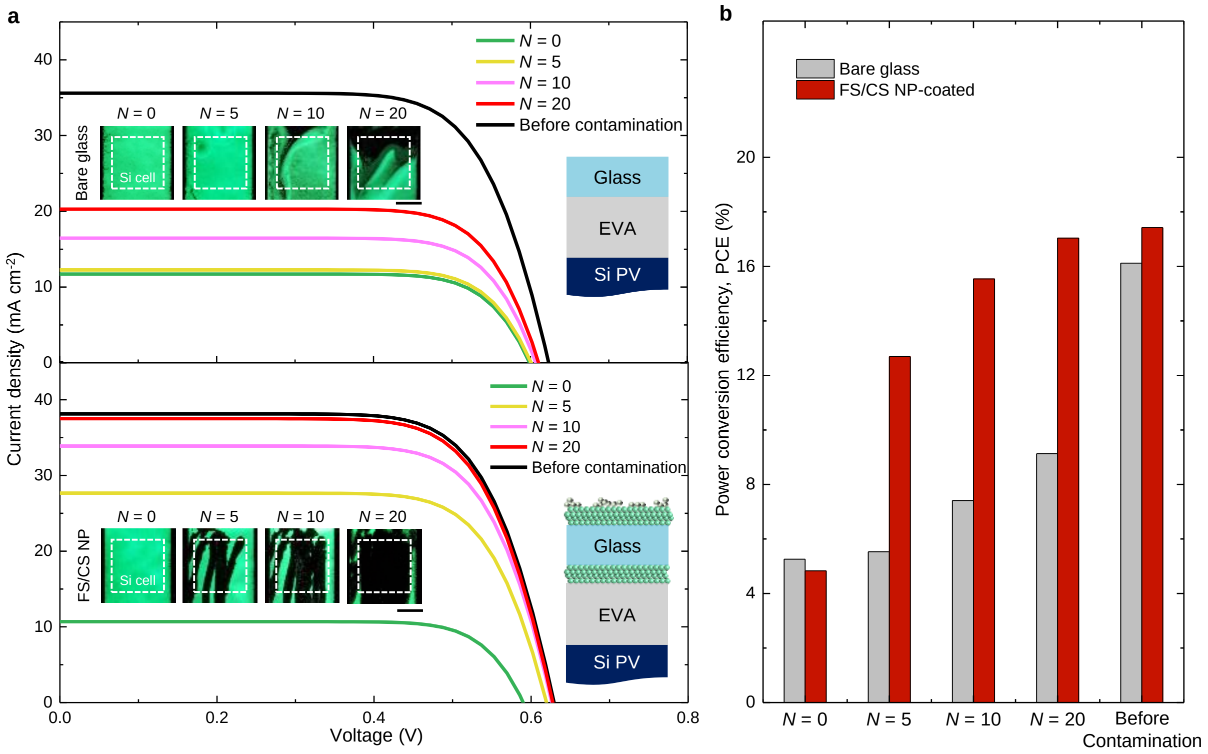

Figure S12. Photovoltaic experiments for small-sized contaminations. a) Measured *J–V* curves of PV device with bare (upper) and FS/CS NP-coated glass (lower) samples for *N* (0, 5, 10, and 20) water droplets. Insets: structure of silicon solar cell. Scale bars: 1 cm. b) PCE values of PV device for bare and FS/CS NP-coated glass samples in (a). Inset: images obtained for *N* (0, 5, 10, and 20) water droplets after self-cleaning.

13. AFM and SEM images after reliability tests

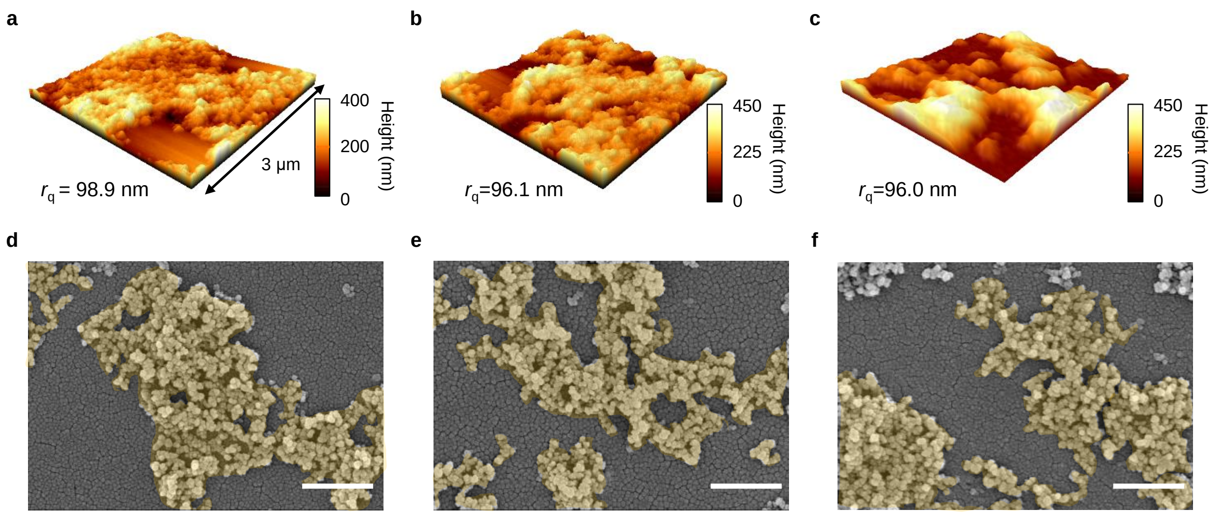

Figure S13. AFM and SEM images after reliability tests. a–f) AFM (a–c) and SEM (d–f) images after exposure of accelerated environmental stress coupled with UV irradiation and high ambient temperature environment (a,d), after water droplet impacts on the sample (b,e) and after repetitive thermal stress (c,f), respectively. The false-colored areas indicate the FS NP coating. Scale bars: 500 nm.

14. Defrosting experiments

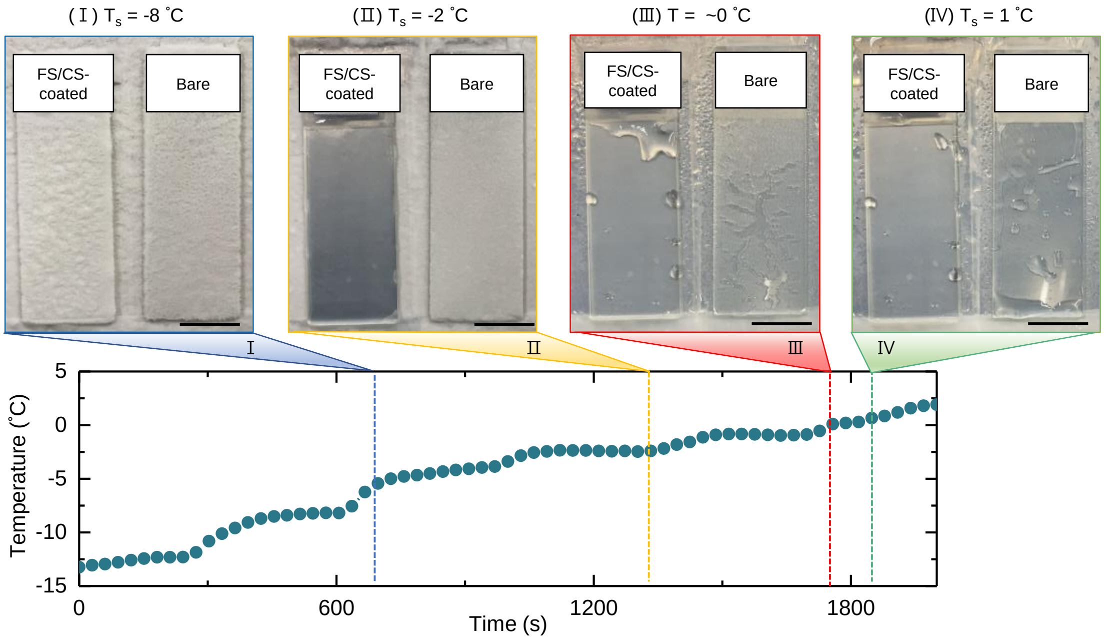

Figure S14. Defrosting experiments. Photo images of FS/CS NP-coated and bare glasses obtained at the surface temperatures of -8 °C (labeled I), -2 °C (labeled II), 0 °C (labeled III) and 1 °C (labeled IV), respectively (upper). The measured temporal surface temperatures (T_s) are plotted together (lower). Scale bars: 1 cm.

15. Previously reported transparent, superhydrophobic surfaces.

Table S1. Previously reported transparent, superhydrophobic surfaces

Structure		Reference	Visible transmittance (%)	Static contact angle (θ_s)	Sliding angle (θ_{sa})
Top-down	Superwavelength (micro)	[6]	85.5	165°	N/A
		[7]	77.0	179°	N/A
	Subwavelength (nano)	[8]	95.4	150°	6°
		[9]	94.7	156°	N/A
		[10]	94.7	172°	N/A
	Hybrid (micro-nano)	[11]	92.4	165°	<1°
		[12]	95.7	139°	N/A
		[13]	94.2	155°	<1°
		[14]	91.6	162°	N/A
Bottom-up	NP clusters (Agglomerated)	[15]	92.4	168°	2°
		[16]	76.0	159°	<1°
		[17]	87.5	156°	6°
		[18]	85.0	159°	7°
		[19]	75.0	160°	<1°
	NP clusters (Aggregated)	[20]	87.0	161°	<1°
		[21]	91.8	160°	N/A
		[22]	85.6	155°	<2°
		[23]	95.2	158°	<1°
		[24]	90.6	160°	<5°
		[25]	93.3	165°	<5°
		[26]	92.2	168°	<2°
	Close-packed NPs	[27]	94.8	152°	40°
		[28]	94.4	159°	~10°
		[29]	97.5	147°	<10°

16. Contact angles of hydrophobically-treated CS NP and FS/CS NP coatings.

Table S2. Contact angles of hydrophobically-treated CS NP and FS/CS NP coatings

Structure	Advancing contact angle (θ_a)	Static contact angle (θ_s)	Receding contact angle (θ_r)	Contact angle hysteresis ($\Delta\theta$)
CS NP-coated	132.0 ± 1.7°	121.7 ± 2.2°	96.9 ± 1.8°	35.1± 1.7°
FS/CS NP-coated	170.6 ± 2.9°	170.1 ± 3.3°	165.5 ± 1.8°	5.1 ± 2.6°
